# EA^3^: A softmax algorithm for evidence appraisal aggregation

**DOI:** 10.1371/journal.pone.0253057

**Published:** 2021-06-17

**Authors:** Francesco De Pretis, Jürgen Landes

**Affiliations:** 1 Department of Biomedical Sciences and Public Health, School of Medicine and Surgery, Marche Polytechnic University, Ancona, Italy; 2 Department of Communication and Economics, University of Modena and Reggio Emilia, Reggio Emilia, Italy; 3 Munich Center for Mathematical Philosophy, Ludwig-Maximilians-Universität München, München, Germany; Fuzhou University, CHINA

## Abstract

Real World Evidence (RWE) and its uses are playing a growing role in medical research and inference. Prominently, the 21st Century Cures Act—approved in 2016 by the US Congress—permits the introduction of RWE for the purpose of risk-benefit assessments of medical interventions. However, appraising the quality of RWE and determining its inferential strength are, more often than not, thorny problems, because evidence production methodologies may suffer from multiple imperfections. The problem arises to aggregate multiple appraised imperfections and perform inference with RWE. In this article, we thus develop an evidence appraisal aggregation algorithm called EA^3^. Our algorithm employs the softmax function—a generalisation of the logistic function to multiple dimensions—which is popular in several fields: statistics, mathematical physics and artificial intelligence. We prove that EA^3^ has a number of desirable properties for appraising RWE and we show how the aggregated evidence appraisals computed by EA^3^ can support causal inferences based on RWE within a Bayesian decision making framework. We also discuss features and limitations of our approach and how to overcome some shortcomings. We conclude with a look ahead at the use of RWE.

## Introduction

Real World Evidence (RWE) [1] is one of the new frontiers of medical research and inference and attracts growing interests in academic and industrial research. RWE comprises observational data obtained outside the context of Randomised Controlled Trials (RCTs) which are produced during routine clinical practice. According to a broader understanding, it may be possible to point at any source of information, that is related to medications and not directly retrievable from RCTs, as a potential generator of RWE, e.g. social networks [2].

Despite being known for a long time and in some cases applied as an informative support in the drug approval process [3] (e.g. the anticoagulant Rivaroxaban [4]), RWE has recently been brought to the fore by the US Congress with the Pub.L. 114—255 (21st Century Cures Act) which modified in 2016 the Food and Drug Administration (FDA) procedures for medications licensing. The act allows, under certain conditions, pharmaceutical companies to provide “data summaries” and RWE such as observational studies, insurance claims data, patient input, and anecdotal data rather than RCTs data for drug approval purposes. After the turn to RCTs as gold-standard in the drug approval process, this is the first act allowing for uses of RWE in the drug approval process in an industrialised country. This move sparked interest also of the European Medical Agency (EMA) and the Japanese Pharmaceuticals and Medical Devices Agency (PMDA) [5, 6].

The use and standards for proper use of RWE have ignited a serious debate in the scientific community [7–11]; for a special issue see [12]. Proponents of the use of RWE point to the fact that RWE can be produced much faster than conducting and analysing a clinical study [13, 14]. This allows pharmaceutical companies to obtain approval for new products or new indications (off-label use) quicker, which can benefit companies as well as patients [15]. Faster and safe drug approval procedures are particularly relevant during the current Covid-19 pandemic [16, 17]. However, many researchers have expressed concerns related to data quality, validity, reliability and sensitivity to capture the exposure, adverse effects and outcomes of interest when using RWE [18–22]. Using RWE for medical inference presents methodological challenges [23], though some efforts have been carried out to efficiently merge evidence coming from RCTs and observational studies [24–26], also for causal inference purposes [27, 28]. Attempts to provide a framework for appraising the quality of evidence for medical inference have been going on since long before the current debate on uses of RWE began, e.g. GRADE [29, 30]. However, these frameworks do not provide a clear way to quantitatively solve this problem nor do they lend themselves to an integration into a standard decision making framework [31–34].

The US National Research Council has issued following call: “*The risk-of-bias assessment of individual studies should be carried forward and incorporated into the evaluation of evidence among data streams*” [35]. This point appears crucial to us for appraising RWE. There is however no commonly accepted methodology for carrying out RWE appraisals. A possible solution to this problem is to split the appraisal of RWE into multiple more manageable appraisals along different dimensions and then to aggregate these appraisals. However, how can we aggregate these multiple appraisals? Subsequently, how can we use this aggregate for decision making?

We here address these two questions by proposing an algorithm based on (1) the softmax function—a generalisation of the logistic function to multiple dimensions—as an instrumental tool for aggregation within (2) a Bayesian decision making framework. While the softmax function was initially introduced in statistical mechanics, it has now found wide-spread applications in machine learning and artificial intelligence methods at large [36–38]. On the other hand, Bayesian approaches are increasing in popularity in part due to their intuitive incorporation of information and updating procedures.

Drawing on these traditions, we present an **E**vidence **A**ppraisal **A**ggregation **A**lgorithm, EA^3^ (suggested pronunciation: “EA-cube”) compressing a generic vector of evidence appraisals along multiple dimensions into a scalar. Roughly, input data (evidence appraisals) are first processed through the softmax function and next aggregated by the application of a geometric mean. EA^3^ is then shown to have some desirable properties. It offers the possibility of emphasizing or the de-emphasizing the maximum values associated to each evidence appraisal via a cautiousness parameter (the thermodynamic *β* of softmax). Furthermore, EA^3^ allows one to incorporate the importance of the dimensions of appraisals. Eventually, we show how EA^3^ can be used to support assessments of causal hypotheses within a Bayesian decision making approach.

To the best of our knowledge, EA^3^ represents one of the first attempts to solve the problem of evidence appraisal through an easy-to-exploit numerical measure [39, 40]. In line with the previously mentioned US Environmental Protection Agency (EPA) recommendations [35], our appraisals can be understood as risk-of-bias assessments—but also of other possible methodological flaws. We offer a formalisation of such assessments and facilitate a tracking of these assessments through evidence aggregation to the calculation of probabilities of hypotheses of interest. Our proposal commits to be thus “*transparent, reproducible and scientifically defensible*” as suggested by the EPA [35, p. 79].

The rest of this article is organised as follows: in **Materials and Methods**, we introduce the softmax function as well as a motivating example and then present our softmax algorithm in some detail and discuss its properties. The **Results** section puts forward a method to apply EA^3^ in Bayesian decision making problems. A final **Discussion** outlines advantages and limitations of our approach and points to important future work.

## Materials and methods

In this section, we first introduce the softmax function, then we present the EA^3^ algorithm and discuss its properties.

### Softmax

The softmax function (more correctly softargmax, also known as normalised exponential function) is a function from $R^{k}$ to $R^{k}$ (k∈N) mapping a vector $\vec{A}=\langle a_{1}\ldots a_{l}\ldots a_{k}\rangle \in R^{k}$ (*k* ≥ 2) to a vector $\sigma (\vec{A})$ as follows:

$$\begin{array}{c} \sigma {\left(\vec{A}\right)}_{l}=\frac{\text{exp}\left(\beta a_{l}\right)}{\sum_{i=1}^{k}\text{exp}\left(\beta a_{i}\right)}\;, \end{array}$$ (1)

where *β* is a real number different from zero, see Table 1 for an overview of key notation. We now briefly discuss some of the properties of the softmax function (henceforth softmax) and recall some of its applications to mathematical physics, probability theory, statistics, machine learning and artificial intelligence.

**Table 1:** Key notation.

*k*	number of dimensions of appraisals
$\vec{A}$	tuple of *k*-appraisals
$\vec{R}$	ranking of dimensions of appraisal
$\vec{A}\times \vec{R}$	weighted mean of $\vec{A}$ and $\vec{R}$
*β*	cautiousness parameter
*v*_*f*_	appraisal aggregate output of EA^3^
EA^3^($\vec{A}$, $\vec{R}$)	appraisal aggregate output of EA^3^ applied to appraisals $\vec{A}$ and ranking $\vec{R}$
*c*@*k*	k-tuple with all entries equal to *c*
Ω	finite set of possible worlds
E	body of evidence
*P*	probability function
*Q*	probability function when RWE is not taken at face value
©	drug *D* causes adverse reaction *E*
Ind	indicator of causation
RoG	Rate of Growth indicator of causation

#### Normalisation

While the input vector may contain any real number, the output of softmax is normalised in the sense that all components of the output vector are in the unit interval and sum to one. The output vector can hence be understood as a probability distribution over *k* elementary events where the probabilities are proportional to the exponential of the input vector.

#### Translational invariance

Softmax is invariant under translations: let ${\vec{A}}^{\prime }$ be obtained from a vector $\vec{A}$ by adding a constant c∈R to every component of *A* then

$$\begin{array}{cc} \sigma {\left({\vec{A}}^{\prime }\right)}_{l} & =\frac{\text{exp}\left(\beta \left(a_{l}+c\right)\right)}{\sum_{i=1}^{k}\text{exp}\left(\beta \left(a_{i}+c\right)\right)}=\frac{\text{exp}\left(\beta a_{l}\right)\cdot \text{exp}\left(\beta c\right)}{\sum_{i=1}^{k}\text{exp}\left(\beta a_{i}\right)\cdot \text{exp}\left(\beta c\right)}\\ & =\sigma {\left(\vec{A}\right)}_{l}\;. \end{array}$$

So, if ${\vec{A}}^{\prime }$ is obtained from $\vec{A}$ via translation, then $\sigma \left({\vec{A}}^{\prime }\right)=\sigma \left(\vec{A}\right)$.

Softmax is **not scale invariant**. It is easy to prove that multiplying every component of an input vector $\vec{A}$ by some constant *c* does, in general, not return the same output vector.

**The** *β* **parameter** allows one to change the base of the exponential function. This choice permits one to emphasise or de-emphasise the maximum value belonging to the input vector, the greater *β* the greater the maximal component of the output vector. For *β* = +∞ the output vector vanishes everywhere except those components at which the input vector is the greatest (in this case, softmax becomes an *argmax*). Conversely, for *β* = −∞ the output vector vanishes everywhere except those components at which the input vector is the smallest (*argmin*). In the limit case *β* = 0 the output vector is the uniform probability distribution resulting in a loss of all the information contained in the input.

The first use of softmax goes back to 1868 when Ludwig Boltzmann introduced the function for modelling ideal gases. Today, softmax is known as the Boltzmann-Gibbs distribution in statistical mechanics, where the index set {1, …, *l*, …, *k*} represents the microstates of a classical thermodynamic system in thermal equilibrium and *a*_*l*_ is the energy of that state *l* and *β* the inverse temperature (thermodynamic *β*) [41, 42]. Beyond the representation of physical systems, the distribution and this modeling have paved the way to some noteworthy algorithms based on the same statistical mechanics assumptions, e.g. Gibbs sampling [43].

The normalisation property has led to applications of softmax in probability theory to represent a categorical distribution [44] and in statistics to define a classification method through the so-called softmax regression, an equivalent to multinomial logistic regression [45, 46]. This property has been widely used also in medical statistics [47–49].

In recent years, two fields have been seeing a raising interest towards softmax: machine learning and artificial intelligence [50, 51]. The term softmax itself has been first introduced by Bridle in neural networks, where it is usually employed as an activation function to normalise data [52]. In computer science, applications of softmax are varied: classification methods (again, softmax regression) for supervised and unsupervised learning [53–55], computer vision [56–58], reinforcement learning [59–61] and hardware design [62], just to name some current areas of application. Additionally, a considerable number of conference papers is witnessing the popularity of softmax and its proposed variants [63–67].

### Motivating example

Consider the hypothesis that paracetamol use causes asthma in children [68]. Only relatively few RCTs have been conducted that could help us determine the truth of this hypothesis [69]. RWE will thus have to (!) play an important role in treatment and prescription decisions that have to be made now, that is before (meta-analyses of) RCTs can deliver conclusive evidence [70].

RWE for and against this causal hypothesis is, for example, obtained from relatively large surveys [71–77]. Such evidence is clearly less confirmatory than well-run RCTs and we hence need to find a way to appraise this evidence. De Pretis et al. (2019) [78] suggested that such surveys can be appraised along three independent and relevant dimensions: duration of the surveyed time period, the sample size and the methodology for adjustment and stratification. Appraisals are represented by numbers in the unit interval where 1 represents a perfect appraisal (e.g, perfect methodology for adjustment and stratification) and 0 represents the worst possible score (e.g. tiny sample size). These three appraisals are then aggregated by taking their arithmetic mean.

Simply taking the arithmetic mean is problematic for a number of reasons. Firstly, the dimensions of appraisal are all given the same weight. This problem can be easily addressed by moving to a weighted mean where the weights represent the importance of the dimensions of appraisal. Secondly, every weighted mean of three equal numbers *c* is equal to *c*. That is, multiple imperfections of RWE of equal degree *c* lead to an overall appraisal equal to *c*. We think, the overall appraisal ought to be less than *c*, multiple imperfections are worse than just one imperfection. Thirdly, a decision maker has no flexibility in the aggregation of appraisal to represent his/her attitude towards the question “how much worse are multiple imperfections than a single imperfection”. We hence think that a suitable aggregation is not idempotent.

We next present and explain the EA^3^ algorithm to aggregate evidence appraisals, which addresses these points.

### The evidence appraisal aggregation algorithm EA^3^

We assume that evidence is appraised in *k* relevant and pairwise different and mutually independent dimensions represented by a normalised appraisal vector $\vec{A}=\langle a_{1}\ldots a_{l}\ldots a_{k}\rangle \in {\left[0,1\right]}^{k}$, see the ***E-Synthesis*** subsection for a suggested set of dimensions for appraisal. We do not commit to a fixed number of evidence appraisals (in agreement with multi criteria decision making in medicine [33] and risk prediction for multiple outcomes [79]).

We also make use of a given ranking of the importance of the different dimensions of appraisal. We represent this ranking by a vector $\vec{R}=\langle r_{1}\ldots r_{l}\ldots r_{k}\rangle \in {\left(0,1\right)}^{k}$ such that $\sum_{i=1}^{k}r_{i}=1$. The more important the appraisal *a*_*l*_, the greater the value *r*_*l*_.

EA^3^ proceeds in 5 steps listed in Table 2 and explained below:

1. Appraisals weighted by ranking:
  $$\begin{array}{c} a_{l}^{\left[1\right]}≔r_{l}\cdot a_{l}\;\text{for}\;\text{all}\;1\leq l\leq k. \end{array}$$
  **Description**. Step 1 weighs every appraisal by its importance.
2. Softmax with a positive thermodynamic *β*
$$\begin{array}{c} a_{l}^{\left[2\right]}≔\frac{\text{exp}(\beta a_{l}^{\left[1\right]})}{\sum_{i=1}^{k}\text{exp}\left(\beta a_{i}^{\left[1\right]}\right)}=\frac{\text{exp}(\beta \cdot r_{l}\cdot a_{l})}{\sum_{i=1}^{k}\text{exp}\left(\beta \cdot r_{i}\cdot a_{i}\right)} \end{array}$$
  **Description**. Step 2 applies, as advertised above, softmax with a parameter *β* representing cautiousness, cf. the discussion following Proposition 1.
3. Rescaling
$$\begin{array}{c} a_{l}^{\left[3\right]}≔a_{l}^{\left[2\right]}\cdot \sum_{i=1}^{k}r_{i}\cdot a_{i}=\frac{\text{exp}(\beta \cdot r_{l}\cdot a_{l})}{\sum_{i=1}^{k}\text{exp}\left(\beta \cdot r_{i}\cdot a_{i}\right)}\cdot \left(\vec{A}\times \vec{R}\right), \end{array}$$
  where × denotes the scalar product between two vectors of the same length *k*.
  **Description**. Step 3 rescales the softmax of Step 2 by aggregated ranked appraisals. Softmax has the well-known property that it is invariant under uniform pointwise translations, *σ*(〈*a*_1_, …, *a*_*k*_) = *σ*(〈*a*_1_ + *c*, …, *a*_*k*_ + *c*〉). This property means for our application that applying softmax to a study *S*_1_ and to a study *S*_2_ which is appraised to be better according to every dimension by the same amount (*c*) it holds that *σ*(*S*_1_) = *σ*(*S*_2_). This is clearly undesirable as a uniformly better study should score better than a uniformly worse study. Multiplying by $\vec{A}\times \vec{R}$ is a simple and intuitive way of ensuring that EA^3^ is not invariant under uniform pointwise translations. Not only is our algorithm sensitive to pointwise translations, it is even the case that every improvement of an appraisal leads to a greater number *v*_*f*_ (see Proposition 2).
4. Geometric averaging:
  $$\begin{array}{cc} v: & =\frac{\prod_{i=1}^{k}\text{exp}\left(\beta \cdot r_{i}\cdot a_{i}\right)}{\sum_{i=1}^{k}\text{exp}\left(\beta \cdot r_{i}\cdot a_{i}\right)}\cdot \left(\vec{A}\times \vec{R}\right)=\frac{\text{exp}(\beta \cdot \left(\vec{A}\times \vec{R}\right))}{\sum_{i=1}^{k}\text{exp}\left(\beta \cdot r_{i}\cdot a_{i}\right)}\cdot \left(\vec{A}\times \vec{R}\right) \end{array}$$
  **Description**. Step 4 compresses the vector to a scalar. To achieve this task, we apply a geometric mean, as it is routinely performed in machine learning for comparing items with a different number of properties and numerical ranges [80–82].
5. Normalisation to unit interval:
  $$v_{f}≔v\cdot \frac{\sum_{i=1}^{k}\text{exp}\left(\beta \cdot r_{i}\right)}{\text{exp}(\beta )}=\frac{\text{exp}(\beta \cdot \left(\vec{A}\times \vec{R}\right))}{\text{exp}(\beta )}\cdot \frac{\sum_{i=1}^{k}\text{exp}\left(\beta \cdot r_{i}\right)}{\sum_{i=1}^{k}\text{exp}\left(\beta \cdot r_{i}\cdot a_{i}\right)}\cdot \left(\vec{A}\times \vec{R}\right)$$
  **Description**. Step 5 ensures that the final output is in the unit interval. We find this normalisation convenient for our application and point out that this step might not be necessary for other applications.

**Table 2:** EA^3^ algorithm structure with objectives described for each step.

Step	Objective
1	Appraisals weighted by ranking
2	Softmax with a positive thermodynamic *β*
3	Rescaling
4	Geometric averaging
5	Normalisation to unit interval

To summarize, given two *k*-tuples $\vec{A},\vec{R}\in {\left[0,1\right]}^{k}\times {\left(0,1\right)}^{k}$ as input the algorithm returns a single number in the unit interval as output. We can understand EA^3^ as a map and thus write EA^3^($\vec{A}$, $\vec{R}$) ∈ [0, 1] (see Corollary 1 for a proof that EA^3^ maps into the unit interval).

### Properties of EA^3^

Denoting by *c*@*k* a vector of length *k* with all components equal to *c*, we find that:

**Proposition 1**. *EA*^3^ *is not idempotent, i.e. for all c* ∈ [0, 1] *and all β* > 0 *it holds that*

$$\begin{array}{c} {\text{EA}}^{3}\left(c@k,\frac{1}{k}@k\right)=c\cdot \text{exp}(\beta \cdot \left(c-1\right)\cdot \left(1-\frac{1}{k}\right))\;. \end{array}$$ (2)

*Proof*. The computation is straightforward:

$$\begin{array}{cc} v_{f}: & =\frac{\text{exp}(\beta \cdot \left(c@k\times \frac{1}{k}@k\right))}{\text{exp}(\beta )}\cdot \frac{\sum_{i=1}^{k}\text{exp}\left(\beta \cdot \frac{1}{k}\right)}{\sum_{i=1}^{k}\text{exp}\left(\beta \cdot \frac{c}{k}\right)}\cdot \left(c@k\times \frac{1}{k}@k\right)\\ & =\frac{\text{exp}(\beta \cdot c)}{\text{exp}(\beta )}\cdot \frac{\text{exp}(\beta \cdot \frac{1}{k})}{\text{exp}(\beta \cdot \frac{c}{k})}\cdot c\\ & =\text{exp}\left(\beta \cdot \left(c-1\right)\right)\cdot \frac{1}{\text{exp}(\left(c-1\right)\cdot \frac{\beta }{k})}\cdot c\\ & =c\cdot \text{exp}(\beta \cdot \left(c-1\right)\cdot \left(1-\frac{1}{k}\right))\;. \end{array}$$

**Fig 1:**
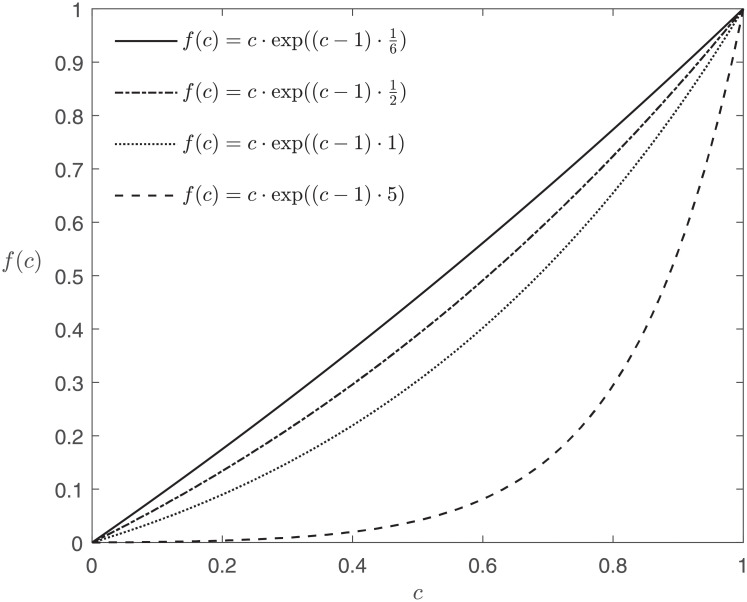
Behaviour of ${\text{EA}}^{3}\left(c@k,\frac{1}{k}@k\right)$ for varying *β*, where $\beta \cdot (1-\frac{1}{k})$ is the second factor within the scope of the exponential function. The smaller parameter *β* and the greater the number of appraisals (the greater *k*), the closer ${\text{EA}}^{3}\left(c@k,\frac{1}{k}@k\right)$ gets to the identity map. This graph clearly displays the monotonicity of these functions.

This observation demonstrates the role of *β* and how the simplest ranking scheme (all dimensions are ranked equally) acts in the simple case in which all appraisals are equal to *c*, see Fig 1 for an illustration. The greater *β*, the smaller *v*_*f*_, the further away the curves plotted in Fig 1 are away from the identity map. This means that a study with all appraisals equal to *c* will have an aggregate, *v*_*f*_, equal to less than *c*. In other words, RWE that is less than perfect in more than one respect has an even lower aggregated appraisal. This seems right, studies which might produce poor evidence for multiple reasons are considered to produce very poor evidence. It is for this reason that we require that *β* > 0.

*β* = +∞ represents maximal cautiousness, if the study is not perfect in all respects (*c* < 1), then ${\text{EA}}^{3}\left(c@k,\frac{1}{k}@k\right)=0$. *β* = 0 represents maximal optimism (and in our eyes overly strong optimism) in that ${\text{EA}}^{3}\left(c@k,\frac{1}{k}@k\right)=c$, a study with a number of imperfections (*c* < 1) is overall as good as just a single imperfection.

Furthermore, note that if *β* ≪ 0, then Eq (2) may exceed 1. So, in such a case our Step 5 would fail normalise *v*_*f*_ to the unit interval and a different normalisation step would be required.

**Definition 1** (Monotonicity) *We call a function* $f:{\left[0,1\right]}^{k}\to R$ monotone, *if and only if the restriction of f to all coordinates is a strictly monotonously increasing function*.

**Proposition 2**. *For every given fixed ranking scheme* $\vec{R}$, *the function EA*^3^(·, $\vec{R}$) *is monotone*.

This proposition is key for our purposes as it states that every improved appraisal entails a better aggregate. In other words, better methodologies have a greater *v*_*f*_ which in turn have greater (dis-)confirmatory weight (see the Results section).

*Proof*. It suffices to verify that all the partial derivatives of EA^3^(·, $\vec{R}$) with respect to the *a*_*l*_ are strictly positive for all *a*_*l*_ ∈ [0, 1]. Since the normalisation step is a multiplication by a scalar which does not depend on $\vec{A}$, it suffices to verify that all the partial derivatives of *v* with respect to the *a*_*l*_ terms are strictly positive for all *a*_*l*_ ∈ [0, 1].

We now compute that this is indeed the case:

$$\begin{array}{c} \frac{\partial }{\partial a_{l}}v=\text{exp}\left(\beta \cdot \left(\vec{A}\times \vec{R}\right)\right)\cdot \frac{\left(a_{l}+\beta \cdot r_{l}\right)\cdot \left(\sum_{i=1}^{k}\text{exp}\left(\beta \cdot r_{i}\cdot a_{i}\right)\right)-\left(\beta \cdot r_{l}\right)\cdot \left(\vec{A}\times \vec{R}\right)}{{\left(\sum_{i=1}^{k}\text{exp}\left(\beta \cdot r_{i}\cdot a_{i}\right)\right)}^{2}}\\ \;=\text{exp}\left(\beta \cdot \left(\vec{A}\times \vec{R}\right)\right)\cdot \frac{\left(a_{l}+\beta \cdot r_{l}\right)\cdot \left(\sum_{i=1}^{k}\text{exp}\left(\beta \cdot r_{i}\cdot a_{i}\right)\right)-\left(\beta \cdot r_{l}\right)\cdot \left(\sum_{i=1}^{k}a_{i}\cdot r_{i}\right)}{{\left(\sum_{i=1}^{k}\text{exp}\left(\beta \cdot r_{i}\cdot a_{i}\right)\right)}^{2}}\\ \;>\text{exp}\left(\beta \cdot \left(\vec{A}\times \vec{R}\right)\right)\cdot \frac{\left(a_{l}+\beta \cdot r_{l}\right)\cdot \left(\sum_{i=1}^{k}r_{i}\cdot a_{i}\right)-\left(\beta \cdot r_{l}\right)\cdot \left(\sum_{i=1}^{k}a_{i}\cdot r_{i}\right)}{{\left(\sum_{i=1}^{k}\text{exp}\left(\beta \cdot r_{i}\cdot a_{i}\right)\right)}^{2}}\\ \;\geq 0\;. \end{array}$$

The sharp inequality follow from the fact that *exp*(*β* ⋅ *x*) > 1 ≥ *x* for all *x* ∈ [0, 1] and all *β* > 0.

**Corollary 1**. *For every given fixed ranking scheme* $\vec{R}$, *the function EA*^3^(⋅, $\vec{R}$) *maps into the unit interval*, [0, 1]. *Furthermore, we note that* $v_{f}\left(\vec{A},\vec{R}\right)=0\Leftrightarrow \vec{A}=0@k$ *and* $v_{f}\left(\vec{A},\vec{R}\right)=1\Leftrightarrow \vec{A}=1@k$.

*Proof*. Applying Proposition 2 it suffices to show that EA^3^(0@*k*, $\vec{R}$) = 0 and EA^3^(1@*k*, $\vec{R}$) = 1. The first condition follows from $0@k\times \vec{R}=0$ and the second from $1@k\times \vec{R}=1$.

Also note that if $\vec{A}=0@k$, then $\vec{A}\times \vec{R}=0$ and thus *v*_*f*_ = 0. If $\vec{A}\neq 0@k$, then $\vec{A}\times \vec{R}>0$ and thus *v*_*f*_ > 0.

Similarly, if $\vec{A}=1@k$, then $\vec{A}\times \vec{R}=1$ and thus *v*_*f*_ = 1. If $\vec{A}\neq 1@k$, then $\vec{A}\times \vec{R}<1$ and thus *v*_*f*_ < 1.

### The motivating example—reconsidered

**Fig 2:**
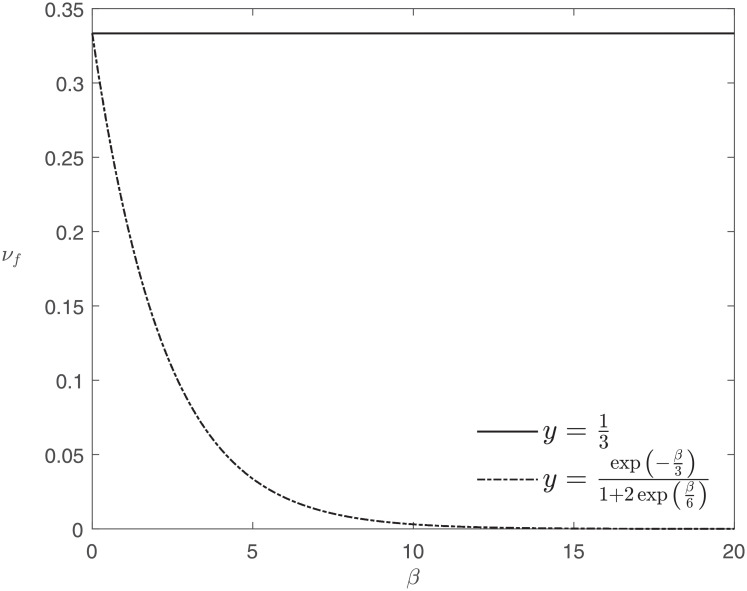
Aggregated appraisals of Karimi et al. (2006) [75] according to De Pretis et al. (2019) [78] (solid line) and EA^3^ (dash-dot line). The latter, lower, curve displays the behaviour with respect to the cautiousness parameter *β*. Both curves agree for *β* = 0 where EA^3^ equals the weighted mean.

**Fig 3:**
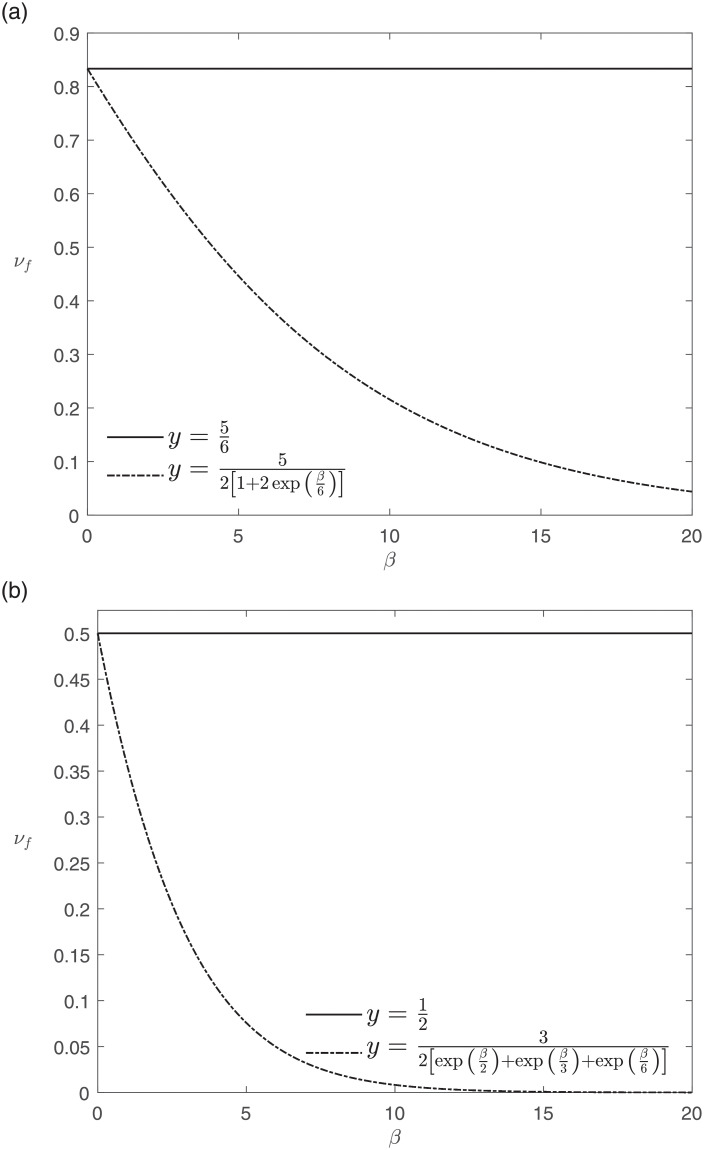
Similarly to Fig 2, the upper panel shows the aggregated appraisals of Newson et al. (2000) [72], Amberbir et al. (2011) [76] and Beasley et al. (2011) [77] according to De Pretis et al. (2019) [78] (solid line) and EA^3^ (dash-dot line). The lower panel depicts the aggregated appraisals for Lesko and Mitchell (1999) [71] and Lesko et al. (2002) [73].

Returning to the suspected causal link between paracetamol use and asthma, we now compare the aggregated appraisals of several RWE-providing surveys involving children, previously considered in [78], according to De Pretis et al. (2019) [78] and according to EA^3^. See Table 3 for the formulae and Figs 2 and 3 for a graphical comparison under the assumption of equally important appraisal dimensions, $\vec{R}=\frac{1}{3}@3$. We note that for *β* = 0 both approaches agree and that the aggregate appraisal computed with EA^3^ decreases with increasing cautiousness parameter *β*.

**Table 3:** Evidence Appraisal Aggregation according to De Pretis et al. (2019) [78] and EA^3^ with equally important appraisal dimensions ($\vec{R}=\frac{1}{3}@3$) where *SS* represents the appraised sample size, *D* the appraised duration and *A* represents the appraised adjustment and stratification.

Survey	*SS*	*D*	*A*	De Pretis et al. (2019)	EA^3^
*Lesko and Mitchell (1999)* [71]	1	0	0.5	$\frac{1}{2}$	$\frac{3}{2[\text{exp}(\frac{\beta }{2})+\text{exp}(\frac{\beta }{3})+\text{exp}(\frac{\beta }{6})]}$
*Newson et al. (2000)* [72]	1	0.5	1	$\frac{5}{6}$	$\frac{5}{2[1+2\text{exp}(\frac{\beta }{6})]}$
*Lesko et al. (2002)* [73]	1	0	0.5	$\frac{1}{2}$	$\frac{3}{2[\text{exp}(\frac{\beta }{2})+\text{exp}(\frac{\beta }{3})+\text{exp}(\frac{\beta }{6})]}$
*Shaheen et al. (2002)* [74]	1	1	1	1	1
*Karimi et al. (2006)* [75]	0.5	0	0.5	$\frac{1}{3}$	$\frac{\text{exp}(-\frac{\beta }{3})}{1+2\text{exp}(\frac{\beta }{6})}$
*Amberbir et al. (2011)* [76]	0.5	1	1	$\frac{5}{6}$	$\frac{5}{2[1+2\text{exp}(\frac{\beta }{6})]}$
*Beasley et al. (2011)* [77]	1	0.5	1	$\frac{5}{6}$	$\frac{5}{2[1+2\text{exp}(\frac{\beta }{6})]}$

We are not aware of other approaches of qualitative aggregations of multiple evidence appraisals for medical inference. We hence lack a standard against which to benchmark our proposal. However, there are substantive bodies of literature on aggregating numerically represented judgements and preferences, which, at times, tackle a formally equivalent aggregation problem. A related proposal for medical inference is the GRADE methodology, which puts forward a way to obtain a qualitative confidence rating in hypotheses. The suggestion is to use the lowest confidence ranking for critical outcomes as the aggregate confidence [83]. By contrast, our approach is quantitative and all appraisals contribute to the aggregate.

Another field relevant our work is the current research on Bayesian hierarchical models for aggregation. In the already mentioned [24, 25] such models are employed to combine different study types in meta-analysis and account for bias, with the objective of its correction. Whereas in this article we consider one study and multiple appraisals of bias, the inverse may be considered true in [24]. There, the author employs a bias-correcting Bayesian hierarchical model [84] to combine different study types in meta-analysis. That model is based on a mixture of two random effects distributions, where the first component corresponds to the model of interest and the second component to the hidden bias structure. The resulting model is thus adjusted by the internal validity bias of the studies included in a systematic review.

## Results. Application of EA^3^ to Bayesian decision making problems

### The bayesian framework

We now illustrate how EA^3^ can be incorporated into the Bayesian decision making framework [85], in which decisions are based on all the available evidence [86]. In this framework, a decision maker is facing a decision problem in which a number of possible acts are at his/her disposal. However, the decision maker is unsure about the state of the world and thus adopts a prior probability function defined over a finite set of possible worlds, Ω.

All the available evidence is then used to determine a posterior probability function by conditionalising the prior probability function. In order to represent the decision maker’s preferences all pairs of acts and worlds, the possible outcomes, are assigned a utility value in the real numbers. Normatively correct decisions are those which maximise the decision maker’s expected utilities, where expectations are calculated with respect to the updated probability function [87–89].

One immediate issue in this framework is that it is hard to calculate a posterior probability function. This issue is normally solved by applying Bayes’ Theorem (see the following subsection). Bayes’ Theorem is ubiquitous in Bayesian analyses and it is straight-forwardly applied, if the evidence can be taken at face value. In medical inference, where evidence cannot be taken at face value, numerous methodological design features and choices (conscious and subconscious) bear on the information a study provides.

### Bayes’ Theorem

Consider a set of exhaustive and mutually exclusively statistical hypotheses *H*_1_, …, *H*_*n*_, i.e. the states of the world. Let us denote the available evidence by E. Bayes’ Theorem then allows us to compute the posterior probability of the hypothesis *H*_*h*_

$$\begin{array}{cc} \underset{\text{Posterior}}{\underset{︸}{P(H_{h}|E)}} & =\frac{P(E\wedge H_{h})}{P(E)}=P\left(H_{h}\right)\cdot \frac{P(E\wedge H_{h})}{P\left(H_{h}\right)\cdot P\left(E\right)}\\ & =P\left(H_{h}\right)\cdot \frac{P(E|H_{h})}{P\left(E\wedge H_{1}\right)+P\left(E\wedge H_{2}\right)+\ldots +P\left(E\wedge H_{n}\right)}\\ & =\underset{\text{Prior}}{\underset{︸}{P(H_{h})}}\cdot \frac{P(E|H_{h})}{P\left(E|H_{1}\right)P\left(H_{1}\right)+P\left(E|H_{2}\right)P\left(H_{2}\right)+\ldots +P\left(E|H_{n}\right)P\left(H_{n}\right)}\;. \end{array}$$

So, the posterior probability can be computed from prior probabilities over hypotheses and conditional probabilities. The prior probabilities are provided by the decision maker’s prior beliefs about the state of the world. The conditional probabilities are likelihoods specified by the statistical hypotheses. Hence, computing the posterior probability is a simple exercise in the probability calculus—*under the assumption* that the conditional probabilities are likelihoods specified by statistical models.

In medical inference problems with RWE, the calculations of Bayes’ Theorem remain valid, the statistical models however *do not* specify the relevant likelihoods for RWE. The challenge hence arises to specify these conditional probabilities. We next show how this can be done via an application of EA^3^.

### EA^3^ and posterior probabilities of hypotheses based on a single RWE study

How should the posterior probabilities $Q(E_{1}|H)$ look like, given a single study $E_{1}$? For starters, the evidence can be taken at face value, $\vec{A}=1@k$, then $Q(E_{1}|H)$ should just be $P(E_{1}|H)$. If the evidence contains no information whatsoever, $\vec{A}=0@k$ and *v*_*f*_ = 0, then the posterior $Q(E_{1}|H)$ should just equal the prior probability $P(E_{1})$, so $Q\left(E_{1}|H\right)=P\left(E_{1}\right)$. That is, whether *H* is true or not, this does not change the probability of obtaining $E_{1}$. In all other cases, the posterior probability $Q(E_{1}|H)$ should be somewhere between the posterior $P(E_{1}|H)$ and the prior probability $P(E_{1})$.

These considerations suggest that $Q(E_{1}|H)$ may be computed as a weighted mean of the posterior and the prior probability:

$$\begin{array}{c} Q\left(E_{1}|H\right)=v_{f}\cdot P\left(E_{1}|H\right)+\left(1-v_{f}\right)P\left(E_{1}\right)\;. \end{array}$$ (3)

Applying Corollary 1 we see that $Q(E_{1}|H)$ is different from the prior, if the posterior and the prior are different and *v*_*f*_ > 0.

From a theoretical point of view, one may interpret the convex combination in Eq (3) as a Jeffrey update [90]. Under this interpretation, *v*_*f*_ is interpreted as the probability that the evidence can be taken at face value and 1 − *v*_*f*_ can be interpreted as the probability that the evidence is completely uninformative.

The modified posterior probability of a hypothesis given one available RWE study is

$$\begin{array}{cc} Q(H_{h}|E_{1}) & =\frac{Q\left(H_{h}\right)\cdot Q\left(E_{1}|H_{h}\right)}{Q\left(E_{1}|H_{1}\right)Q\left(H_{1}\right)+Q\left(E_{1}|H_{2}\right)Q\left(H_{2}\right)+\ldots +Q\left(E_{1}|H_{n}\right)Q\left(H_{n}\right)}\\ & =\frac{P\left(H_{h}\right)\cdot Q\left(E_{1}|H_{h}\right)}{Q\left(E_{1}|H_{1}\right)P\left(H_{1}\right)+Q\left(E_{1}|H_{2}\right)P\left(H_{2}\right)+\ldots +Q\left(E_{1}|H_{n}\right)P\left(H_{n}\right)}\\ & =\frac{P\left(H_{h}\right)\cdot \left(v_{f}\cdot P\left(E_{1}|H_{h}\right)+\left(1-v_{f}\right)P\left(E_{1}\right)\right)}{\sum_{g=1}^{n}P\left(H_{g}\right)\cdot \left(v_{f}\cdot P\left(E_{1}|H_{g}\right)+\left(1-v_{f}\right)P\left(E_{1}\right)\right)}\;. \end{array}$$ (4)

### EA^3^ and posterior probabilities of hypotheses based on multiple RWE studies

The assumption of a single available RWE study is, of course, rather unrealistic. We now show how to deal with multiple available RWE studies, $E=\{E_{1},\ldots ,E_{s}\}$. We begin by applying EA^3^ to all every study individually, thus obtaining *s*-many outputs $v_{f}^{1},\ldots ,v_{f}^{s}$.

Under the assumption that the studies have been conducted independently from each other, we can generalise Eq (4) as follows:

$$\begin{array}{cc} Q(H_{h}|E) & =\frac{P\left(H_{h}\right)\cdot Q\left(E|H_{h}\right)}{Q\left(E|H_{1}\right)P\left(H_{1}\right)+Q\left(E|H_{2}\right)P\left(H_{2}\right)+\ldots +Q\left(E|H_{n}\right)P\left(H_{n}\right)}\\ & =\frac{P\left(H_{h}\right)\cdot \prod_{r=1}^{s}\left(v_{f}^{r}\cdot P\left(E_{r}|H_{h}\right)+\left(1-v_{f}^{r}\right)P\left(E_{r}\right)\right)}{\sum_{g=1}^{n}P\left(H_{g}\right)\cdot \prod_{r=1}^{s}\left(v_{f}^{r}\cdot P\left(E_{r}|H_{g}\right)+\left(1-v_{f}^{r}\right)P\left(E_{r}\right)\right)}\;. \end{array}$$

### *E*-*Synthesis*

*E-Synthesis* is a Bayesian framework developed for determining probabilities of particular drugs causing a specific adverse reaction [78, 91–95]. In order to facilitate the inference from real world data to a causal hypothesis a layer of so-called “indicators” has been inserted between the hypothesis of interest and the data. The indicators have been derived from Hill’s Guidelines [96] and serve the role as (probabilistic) testable consequences of the causal hypothesis. Learning that an indicator is true raises the probability of the causal hypothesis to a degree. For example, learning that there is correlation between a drug and an adverse effect does *not* entail that the drug causes an adverse reaction. Nevertheless, the presence of a correlation does increase our suspicion that there indeed might be a causal relationship between a drug and an adverse event.

Evidence for adverse reactions often emerges spontaneously in form of case reports and suspected adverse reactions are often confirmed only from observational data [97]. Such RWE is at a high risk of bias and hence the RWE needs to be appraised. *E-Synthesis* has been designed to incorporate such appraisals of RWE, making their role explicit by formalising them as variables (previously, these variables have been termed “evidential modulator” variables). The following dimensions of appraisal have been suggested within the *E-Synthesis* framework: sample size, duration of the study, degree of sponsorship bias, degree of adjustment for covariates and the degree of analogy between the study population and the studied population. Randomised studies can also be appraised for how well blinding, randomization and placebo control were implemented.

*E-Synthesis* was originally intended for philosophical applications, however it has also recently been developed for more practical matters. As yet, no suggestion has been made of how to aggregate evidence appraisals and how to incorporate these appraisals for decision making. We next show how this can be done for a specific indicator of causation applying EA^3^. Denoting by © the causal hypothesis of a drug *D* causing a specific adverse drug reaction (ADR) and by Ind an indicator variable, we have for the posterior probability of © for RWE, Q(©|E),

$$\begin{array}{cc} Q(©|E) & =\frac{Q(©\wedge E)}{Q(E)}\\ & =\frac{Q\left(©\wedge \text{Ind}\wedge E\right)+Q\left(©\wedge \bar{\text{Ind}}\wedge E\right)}{Q\left(©\wedge \text{Ind}\wedge E\right)+Q\left(©\wedge \bar{\text{Ind}}\wedge E\right)+Q\left(\bar{©}\wedge \text{Ind}\wedge E\right)+Q\left(\bar{©}\wedge \bar{\text{Ind}}\wedge E\right)}\\ & =[1+\frac{Q\left(\bar{©}\wedge \text{Ind}\wedge E\right)+Q\left(\bar{©}\wedge \bar{\text{Ind}}\wedge E\right)}{Q\left(©\wedge \text{Ind}\wedge E\right)+Q\left(©\wedge \bar{\text{Ind}}\wedge E\right)}{]}^{-1}\\ & =[1+\frac{Q\left(\bar{©}\right)\cdot Q\left(\text{Ind}|\bar{©}\right)\cdot Q\left(E|\text{Ind}\right)+Q\left(\bar{©}\right)\cdot Q\left(\bar{\text{Ind}}|\bar{©}\right)\cdot Q\left(E|\bar{\text{Ind}}\right)}{Q\left(©\right)\cdot Q\left(\text{Ind}|©\right)\cdot Q\left(E|\text{Ind}\right)+Q\left(©\right)\cdot Q\left(\bar{\text{Ind}}|©\right)\cdot Q\left(E|\bar{\text{Ind}}\right)}{]}^{-1}\\ & =[1+\frac{P\left(\bar{©}\right)\cdot P\left(\text{Ind}|\bar{©}\right)\cdot Q\left(E|\text{Ind}\right)+P\left(\bar{©}\right)\cdot P\left(\bar{\text{Ind}}|\bar{©}\right)\cdot Q\left(E|\bar{\text{Ind}}\right)}{P\left(©\right)\cdot P\left(\text{Ind}|©\right)\cdot Q\left(E|\text{Ind}\right)+P\left(©\right)\cdot P\left(\bar{\text{Ind}}|©\right)\cdot Q\left(E|\bar{\text{Ind}}\right)}{]}^{-1}. \end{array}$$

This calculation uses the fact that the causal indicator variable mediates the inference from data to the causal hypothesis © in the technical sense that conditionalisation on it renders the data and © independent.

### Motivating example—coda

We now return to the motivating example of determining a probability of the causal hypothesis (©) that paracetamol use causes asthma in children. In the *E-Synthesis* approach, the Beasley et al. (2011) [77] study is informative about the “rate of growth” indicator, so Ind = RoG. The posterior probability of © (given only this study) is thus computed as:

$$Q(©|E_{1})=[1+\frac{P\left(\bar{©}\right)\cdot P\left(\text{RoG}|\bar{©}\right)\cdot Q\left(E_{1}|\text{RoG}\right)+P\left(\bar{©}\right)\cdot P\left(\bar{\text{RoG}}|\bar{©}\right)\cdot Q\left(E_{1}|\bar{\text{RoG}}\right)}{P\left(©\right)\cdot P\left(\text{RoG}|©\right)\cdot Q\left(E_{1}|\text{RoG}\right)+P\left(©\right)\cdot P\left(\bar{\text{RoG}}|©\right)\cdot Q\left(E_{1}|\bar{\text{RoG}}\right)}{]}^{-1}.$$

Using Eq (3) and the suggested conditional probabilities of *P*(RoG|⋅) ($P\left(\text{RoG}|©\right)=26\frac{2}{7}\%\approx 26.3\%>P\left(\text{RoG}|\bar{©}\right)=\frac{3}{7}\%\approx 0.4\%$ [78, p. 3]) this becomes

$$\begin{array}{cc} & \;Q(©|E_{1})=\\ & \;[1+\frac{P\left(\bar{©}\right)\cdot \left[\frac{3}{7}\%\cdot \left(v_{f}\cdot P\left(E_{1}|\text{RoG}\right)+\left(1-v_{f}\right)P\left(E_{1}\right)\right)+99\frac{4}{7}\%\cdot \left(v_{f}\cdot P\left(E_{1}|\bar{\text{RoG}}\right)+\left(1-v_{f}\right)P\left(E_{1}\right)\right)\right]}{P\left(©\right)\cdot [26\frac{2}{7}\%\cdot \left(v_{f}\cdot P\left(E_{1}|\text{RoG}\right)+\left(1-v_{f}\right)P\left(E_{1}\right)\right)+73\frac{5}{7}\%\cdot \left(v_{f}\cdot P\left(E_{1}|\bar{\text{RoG}}\right)+\left(1-v_{f}\right)P\left(E_{1}\right)\right)]}{]}^{-1}. \end{array}$$

[78, p. 11] gives $P(E_{1}|\text{RoG})=0.825$ and $P(E_{1}|\bar{\text{RoG}})=0$ and so

$$\begin{array}{cc} Q(©|E_{1})= & [1+\frac{P\left(\bar{©}\right)\cdot \left[\frac{3}{7}\%\cdot \left(v_{f}\cdot 0.825+\left(1-v_{f}\right)P\left(E_{1}\right)\right)+99\frac{4}{7}\%\cdot \left(1-v_{f}\right)P\left(E_{1}\right)\right]}{P\left(©\right)\cdot [26\frac{2}{7}\%\cdot \left(v_{f}\cdot 0.825+\left(1-v_{f}\right)P\left(E_{1}\right)\right)+73\frac{5}{7}\%\cdot \left(1-v_{f}\right)P\left(E_{1}\right)]}{]}^{-1}\\ = & [1+\frac{P(\bar{©})}{P(©)}\cdot \frac{\frac{3}{7}\%\cdot v_{f}\cdot 0.825+\left(1-v_{f}\right)P\left(E_{1}\right)}{26\frac{2}{7}\%\cdot v_{f}\cdot 0.825+\left(1-v_{f}\right)P\left(E_{1}\right)}{]}^{-1}\;. \end{array}$$

The posterior probability of © given by De Pretis et al. (2019) [78] is instead:

$$\begin{array}{cc} P(©|E_{1}) & =[1+\frac{P(\bar{©})}{P(©)}\cdot \frac{P(\text{RoG}|\bar{©})}{P(\text{RoG}|©)}{]}^{-1}=[1+\frac{P(\text{RoG}\wedge \bar{©})}{P(\text{RoG}\wedge ©)}{]}^{-1}\\ & =\frac{P(\text{RoG}\wedge ©)}{P\left(\text{RoG}\wedge ©\right)+P\left(\text{RoG}\wedge \bar{©}\right)}=P\left(©|\text{RoG}\right)\;. \end{array}$$

We note that in the model of De Pretis et al. (2019) [78] this single study is *conclusive evidence* that RoG holds, i.e. there does exist a strongly increasing dose-response relationship between paracetamol use in children and severe onset of asthma. This probability is

$$P\left(©|E_{1}\right)=P\left(©|\text{RoG}\right)=[1+\frac{P(\bar{©})}{P(©)}\cdot \frac{\frac{3}{7}\%}{26\frac{2}{7}\%}{]}^{-1}=[1+\frac{P(\bar{©})}{P(©)}\cdot \frac{3}{184}{]}^{-1}\;.$$

**Fig 4:**
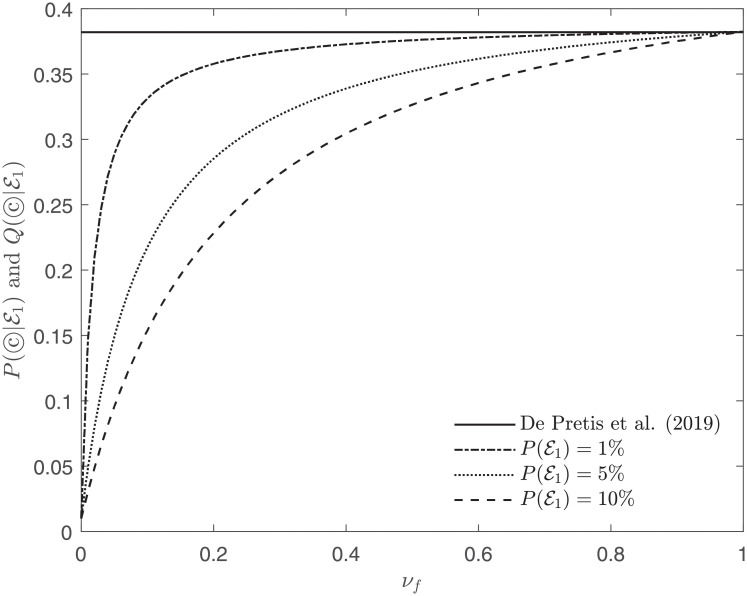
Posterior probability of the causal hypothesis ©, considering Beasley et al. (2011) [77] as evidence $E_{1}$, and computed in agreement with De Pretis et al. (2019) [78] (solid line) and EA^3^ (dash-dot, dotted and dashed lines). For EA^3^, different lines represent different priors $P(E_{1})$, whereas the prior *P*(©) is always set to 1%. All curves agree for *ν*_*f*_ = 1 where De Pretis et al. (2019) [78] becomes a special case of EA^3^.

**Fig 5:**
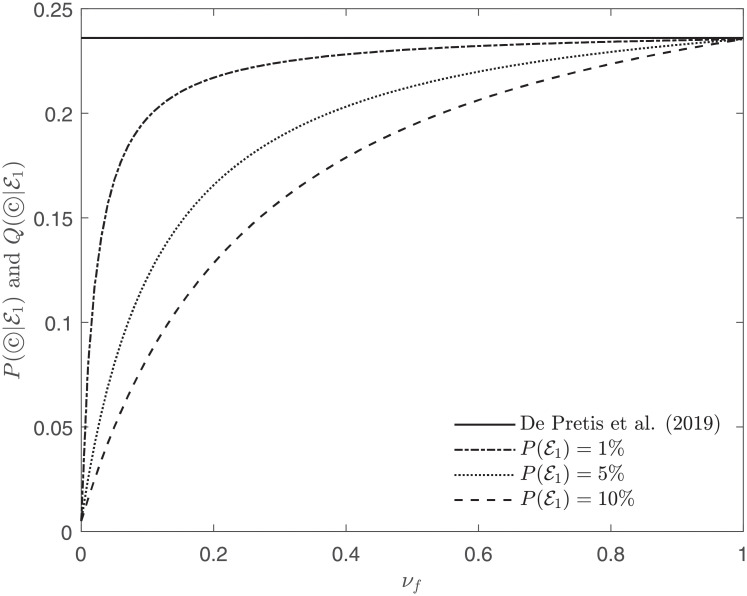
Similarly to Fig 4, the graph pictures the posterior probability of the causal hypothesis ©, considering Beasley et al. (2011) [77] as evidence $E_{1}$, and computed in agreement with De Pretis et al. (2019) [78] (solid line) and EA^3^ (dash-dot, dotted and dashed lines). In this plot, the prior *P*(©) is set to 0.5%.

See Figs 4 and 5 for comparisons of $P(©|E_{1})$ [78] and $Q(©|E_{1})$ (EA^3^).

## Discussion

In this article, we presented an algorithm to support the assessment of the inferential strength of RWE in order to make sound decisions. We proceeded by considering different dimensions of appraisal and then moved on to aggregate multiple appraisals according to the different dimensions into an aggregate. Subsequently, we showed how such an aggregate can be used within a Bayesian decision making framework. Our formal approach carries forward evidence appraisals, incorporates them into an overall appraisal of the evidence and integrates it into decision making [35]. It also enables sensitivity analyses of these appraisals via variation of appraisals, variations of $\vec{A}$, as well as sensitivity analyses of the ranking, variations of $\vec{R}$, and the cautiousness parameter *β*. Furthermore, our approach is transparent, reproducible and scientifically defensible, thus satisfying the desiderata suggested by the US Environmental Protection Agency [35, p. 79].

While our formal aggregation approach is motivated by the need to appraise RWE for medical inference, the developed algorithm is, in principle, applicable to other aggregation problems, too. Whether it is suitable to a particular problem depends on particular circumstances.

Our approach is limited by the assumptions we made, e.g. we assumed that the dimensions of appraisal are independent of each other and that rankings and appraisals can be represented numerically. If at least one of our assumptions fails to hold in an application, then the theoretical considerations made here might not apply. These limitations may be overcome by applications of multi-criteria decision making methodology [98].

In future work, we aim to determine empirically supported dimensions for evidence appraisal, calibrate ranking schemes and determine (normatively and/or descriptively) appropriate values of the *β*-parameter in order to assess the validity and reliability of EA^3^ based on actual data [35]. The *β*-parameter which represents cautiousness reflects risk attitudes which can differ from user to user and from application to application.

Furthermore, EA^3^ reflects the position of a single agent (or of a unanimous committee). In reality, drug approval or withdrawal decisions are a group effort involving experts from different areas (toxicologists, pharmacists, clinicians, statisticians as well as patient representatives [99]), which have different risk attitudes (different *β*), different appraisals (different $\vec{A}$) and different rankings (different $\vec{R}$). We thus plan to integrate EA^3^ into a multi-agent framework which represents different (risk) attitudes, preferences and areas of expertise of stakeholders in drug (un-)safety assessments.

We expect the assessment and use of RWE for medical inference to continue to grow in coming years, drawing on scientific fields in which there are, by the very nature of the investigation, (next to) no randomised studies. For example, in macroeconomics we cannot simply randomly assign countries into different trial arms to learn about the disputed causal relationships between minimum wages and employment [100] and in nutrition science it is not possible to randomise people into drinkers of red wine and non drinkers for a trial lasting several years to learn about the hypothesised causal influences of red wine on health and well-being [101]. Similarly, in pharmacovigilance ADRs may take too long to manifest (years of treatment with olanzapine cause tardive dyskinesia [102]) or be too rare yet fatal (in some cases, 1 fatality in every 10,000 patients [103]) to be detected by RCTs. We think that the use of RWE for pharmacovigilance and medical inference more widely is an area holding great promise despite justified worries about biases and confounding. The development and application of RWE appraisal methods hence seems to become even more important in the future.

## Supporting information

S1 Text (TXT)
